# A Topological Parametric Phonon Oscillator

**DOI:** 10.1002/adma.202309015

**Published:** 2024-10-15

**Authors:** Xiang Xi, Jingwen Ma, Xiankai Sun

**Affiliations:** ^1^ Department of Electronic Engineering The Chinese University of Hong Kong Shatin New Territories Hong Kong

**Keywords:** mechanical metamaterials, nano‐electromechanics, parametric oscillation, strong squeezed interaction, topological Dirac‐vortex states

## Abstract

Topological bosonic systems have recently aroused intense interests in exploring exotic phenomena that have no counterparts in electronic systems. The squeezed bosonic interaction in these systems is particularly interesting, because it can modify the vacuum fluctuations of topological states, drive them into instabilities, and lead to topological parametric oscillators. However, these phenomena remain experimentally elusive because of limited nonlinearities in most existing topological bosonic systems. Here, a topological parametric phonon oscillator is experimentally realized based on a nonlinear nano‐electromechanical Dirac‐vortex cavity with strong squeezed interaction. Specifically, the Dirac‐vortex cavity is parametrically driven to provide phase‐sensitive amplification for topological phonons, leading to the observation of coherent parametric phonon oscillation above the threshold. Additionally, it is confirmed that the random frequency variation caused by fabrication disorders can be suppressed effectively by increasing the cavity size while the free spectral range reduces at a much slower rate, which benefits the realization of large‐area single‐mode lasers. These results represent an important advance in experimental investigations of topological physics with large bosonic nonlinearities and parametric gain.

## Graphical abstract

A topological parametric phonon oscillator is experimentally realized based on a nonlinear nano‐electromechanical Dirac‐vortex cavity with strong squeezed interaction. Fabrication‐disorder‐induced random frequency variation can be suppressed effectively by increasing the cavity size, while the free spectral range reduces at a much slower rate. These results represent an important advance in topological physics with large bosonic nonlinearities and parametric gain.

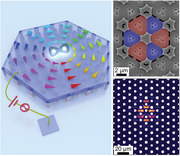

## 1 Introduction

The discovery of topologically nontrivial electronic systems is a significant milestone in condensed matter physics.^[^ ^1^ ^]^ While many topological phases (e.g., quantum Hall, quantum spin Hall, and quantum valley Hall) are based on noninteracting electronic systems, there are intense research activities in exploring topological phases with strong interactions.^[^ ^2^ ^]^ One important example is the topological superconductors, where the nontrivial topology and the fermionic squeezed interaction can jointly lead to the emergence of Majorana fermions.^[^ ^3^ ^]^ Recently, topological physics has been extended to the realms of optics^[^ ^4^, ^5^ ^]^ and acoustics.^[^ ^6^, ^7^ ^]^ The bosonic nature of photons and phonons has aroused intense research interests in investigating topological physics in nonlinear optical or acoustic systems.^[^ ^8^ ^]^ The third‐order nonlinearities in these systems have enabled various important functions or phenomena, including topological third‐harmonic generation,^[^ ^9^, ^10^ ^]^ topological entangled photon‐pair generation,^[^ ^11^, ^12^ ^]^ topological solitons,^[^ ^13^, ^14^ ^]^ and tunable topological states.^[^ ^15^, ^16^ ^]^ On the other hand, the second‐order nonlinearities are also important, because they can provide strong bosonic squeezed interactions between photons or phonons that will unlock new topological phases and functionalities fundamentally different from their counterparts in the electronic domain.^[^ ^17^ ^]^ For instance, parametric oscillators (or parametric lasers) are one of the most important results of bosonic squeezed interactions. They exhibit much lower noises than conventional semiconductor lasers and are widely used as coherent optical/microwave sources for fundamental research and practical applications. While topological theories have recently been applied to conventional semiconductor lasers,^[^ ^18^, ^19^, ^20^, ^21^ ^]^ topological parametric oscillators^[^ ^22^ ^]^ remain experimentally elusive. This is because of negligible second‐order nonlinearities in most existing topological photonic and phononic systems,^[^ ^4^, ^5^, ^6^, ^7^ ^]^ which cannot provide sufficient squeezed interactions.

Dirac‐vortex states are a new type of topological states recently discovered in 2D photonic^[^ ^23^, ^24^ ^]^ and phononic^[^ ^25^, ^26^, ^27^ ^]^ systems. They are described by the Jackiw–Rossi model^[^ ^28^ ^]^ and are mathematically identical to the Majorana fermions in topological superconductors. For conventional 2D systems with an extendable modal area *S*, such as Fabry–Pérot and ring cavities, their free spectral range (FSR) is inversely proportional to *S*, i.e., FSR ∼ 1/*S*. Unlike conventional resonant states in these topologically trivial systems, Dirac‐vortex states with a scalable modal area *S* can have a larger FSR that defies the inverse relationship FSR ∼ 1/*S*. Besides, their resonant frequencies are topologically pinned to the bulk Dirac frequencies and remain nearly constant with respect to *S*. These properties make Dirac‐vortex states an ideal candidate for single‐mode large‐area topological lasers. However, previous experimental demonstrations of Dirac‐vortex states are limited to passive photonic and phononic systems. Despite intense research activities in topological lasers,^[^ ^18^, ^19^ ^]^ lasing or parametric oscillation from the topological Dirac‐vortex states remains to be obtained.

Here, we experimentally demonstrated a topological parametric oscillator from the Dirac‐vortex states on a nonlinear nano‐electromechanical platform. Such nano‐electromechanical systems exhibit strong second‐ and third‐order nonlinearities leading to various phenomena, such as mode cooling,^[^ ^29^ ^]^ vacuum‐state squeezing,^[^ ^30^ ^]^ synchronized oscillating,^[^ ^31^ ^]^ and phonon lasing.^[^ ^32^ ^]^ We harnessed the strong squeezed interaction on this platform for parametric amplification of topological phonons. We observed parametric phonon oscillation above the threshold associated with the linewidth‐narrowing effect. Additionally, we confirmed that the random frequency variation caused by fabrication disorders can be suppressed effectively by increasing the cavity size, while the free spectral range reduces at a much slower rate. Our results provide a robust coherent phonon source for topological integrated phononic circuits, and also represent an important step forward in experimental investigation of the interplay between topology and nonlinearities.

## 2 Results and Discussion

**Figure 1:**
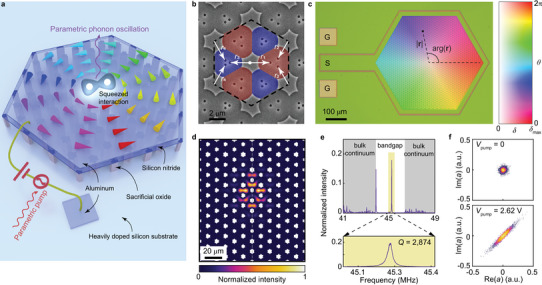
Nano‐electromechanical Dirac‐vortex state with squeezed interaction. a) Conceptual illustration of a nano‐electromechanical Dirac‐vortex parametric phonon oscillator. The device is based on a 2D array of suspended silicon nitride membranes, which encompasses a topological phase winding process rendered by the swirling cones. The heavily doped silicon substrate serves as the electrical ground, and the aluminum layer deposited on silicon nitride is connected to the signal electrode. Applying a combination of a d.c. bias voltage and an a.c. parametric pump voltage across the signal and ground electrodes leads to a strong squeezed interaction for the topological phonons, which enables a Dirac‐vortex parametric phonon oscillator. b) Scanning electron microscope image of the nano‐electromechanical crystal (before aluminum deposition) showing a unit cell of the hexagonal lattice (black dashed hexagon). Each unit cell contains two groups of suspended membranes (colored in blue and red), whose geometries are determined by the positions of the etched holes (*r*_1_, *r*_2_) and (*r*_3_, *r*_4_). c) Optical microscope image of the device (before aluminum deposition). It is color‐coded by the spatially varying parameters *δ*_0_(**r**)  =  *δ*_max_tanh(|**r**|/*R*_0_) and *θ*(**r**)  =  arg(**r**) with *R*_0_/*l*_0_ = 0.5; S, signal electrode; G, ground electrode. d) Simulated intensity profile of the Dirac‐vortex state. e) Measured mechanical intensity spectra of the device in panel (c). The gray‐shaded regions correspond to the bulk continuum. The Dirac‐vortex state lies in the bulk bandgap region with a resonant frequency *ω*_0_/2π = 45.238 MHz and a quality factor ≈2,874. f) Measured vibration quadrature of the Dirac‐vortex state without (*V*_pump_ = 0) and with (*V*_pump_ = 2.62 V, pump frequency = 2*ω*_0_) squeezed interaction.

**Figure** **1**a is a conceptual illustration of a topological Dirac‐vortex parametric phonon oscillator. A 2D array of suspended silicon nitride membranes with a thickness of 150 nm was formed by removing the underlying sacrificial oxide in parts. The suspended membranes can vibrate collectively in the out‐of‐plane direction, and the quantum‐mechanical quantization of the modes of such vibrations is acoustic phonon. A 40‐nm‐thick aluminum layer was deposited on top of silicon nitride to serve as the signal electrode. The heavily doped silicon substrate served as the electrical ground. Figure 1b is a scanning electron microscope image of the fabricated nano‐electromechanical crystal with lattice constant *l*_0_ = 20.8 µm. Small holes (600 nm in diameter) were etched in the silicon nitride layer to enable the penetration of buffered oxide etchant into the sacrificial oxide layer for wet etching purpose (see the “Experimental Section”). Each unit cell contains six nano‐electromechanical membranes, which can be classified into two groups as colored in red and blue. Their geometries are controlled by the relative positions of the small holes (*r*_1_, *r*_2_)  =  (*r*_0_ − *δ*_0_cos*θ* + *δ*_0_sin*θ*, *r*_0_ − *δ*_0_cos*θ* − (*δ*_0_sin*θ*)/2) and (*r*_3_, *r*_4_)  =  (*r*_0_ + *δ*_0_cos*θ* + *δ*_0_sin*θ*, *r*_0_ + *δ*_0_cos*θ* − (*δ*_0_sin*θ*)/2) with *r*_0_  =  5.4 µm. The effective bulk Hamiltonian of the crystal in the linear regime is *H*(**k**) = *v*_D_ · (*σ*_*x*_ *k_x_* + *σ*_*y*_ *k_y_*) − Δ_0_/2 · *σ*_*z*_(*τ*_*x*_cosθ + *τ*_*y*_sinθ), where *σ_x_*, *σ_y_*, *σ_z_*, *τ_x_*, and *τ_y_* are the Pauli matrices, **k** = (*k_x_*, *k_y_*) is the wave vector, Δ_0_ is the bulk bandgap proportional to *δ*_0_, and *v*_D_ is the effective Fermi velocity (see Section S2 in the Supporting Information). As shown in Figure 1c, the device has spatially varying parameters, *δ*_0_(**r**)  =  *δ*_max_tanh(|**r**|/*R*_0_) and *θ*(**r**)  =  arg(**r**), with *δ*_max_ = 300 nm and *R*_0_ controlling the spatially varying bulk bandgap along the radial direction. Such a configuration is mathematically identical to the Jackiw–Rossi model^[^ ^28^ ^]^ and can support the Dirac‐vortex state.^[^ ^27^ ^]^ Theoretically, the modal profile of the Dirac‐vortex state is *f*_0_(**r**) = *g*_0_(|**r**|) · |ψ_0_〉, which is composed of an envelope function *g*_0_(|**r**|) controlling the modal area *S* and a periodic Bloch mode |ψ_0_〉 controlling the modal profile within each unit cell (see Section S2 in the Supporting Information). With such a configuration, the phononic mode of the vibrating membranes acquires the topological properties of the Dirac‐vortex state. Figure 1d shows the simulated intensity profile |*f*_0_(**r**)|^2^ of the Dirac‐vortex state with *R*_0_/*l*_0_ = 0.5; the mode presents a mirror symmetry as the crystal's structure has a mirror symmetry with respect to the gray dashed line in Figure 1d. Figure 1e shows the experimental mechanical intensity spectra for the device directly actuated in the frequency range of 41–49 MHz. In the bulk bandgap region, we can find the topological Dirac‐vortex state with a resonant frequency *ω*_0_/2π = 45.238 MHz and a quality factor ≈2,874 measured at room temperature. The damping rate of the Dirac‐vortex state is *γ*/2π = 15.7 kHz.

Although nonlinear bosonic interaction has been observed in a similar nanomechanical Dirac‐vortex state,^[^ ^27^ ^]^ that device structure cannot provide efficient actuation for strong squeezed interaction. To obtain efficient actuation, we increased the size of the signal electrode substantially by depositing an aluminum layer on the chip covering all the membranes, instead of using a point metal electrode (Figure 1a). With such a configuration, strong squeezed interaction for the Dirac‐vortex state can be obtained by applying a combination of a d.c. bias voltage *V*_0_ and an a.c. parametric pump voltage *V*_pump_cos(2Ω*t*) across the signal and ground electrodes, which exerts an electrocapacitive force

(1) $$F=\frac{\varepsilon {\left[V_{0}+V_{\mathrm{pump}}\cos\left(2\Omega t\right)\right]}^{2}}{2{\left[d-W(r,t)\right]}^{2}}$$

on the nano‐electromechanical membranes (Figure 1a). Here Ω is near the resonant frequency *ω*_0_ of the Dirac‐vortex state with a frequency detuning of Δ = Ω − *ω*_0_, ε is the effective permittivity, *d* is the distance between the silicon substrate and the aluminum layer deposited on silicon nitride, and *W*(**r**,  *t*) = *a*(*t*)*f*_0_(**r**)e^*j*Ω*t*^ + h.c. is the vibrational displacement with *a*(*t*) and *f*_0_(**r**) describing the temporal dynamics and spatial modal profile of the Dirac‐vortex state, respectively. Being dependent on *W*(**r**, *t*), the electrocapacitive force *F* can modify the intrinsic vibrational dynamics of the Dirac‐vortex state. More specifically, the force *F* at the 2Ω frequency can lead to a periodic modulation of the membranes’ spring constant and thus can change the effective damping of the mode at the Ω frequency. Therefore, the parametric pump voltage *V*_pump_cos(2Ω*t*) can lead to degenerate squeezed interaction between the topological Dirac‐vortex phonons. The temporal evolution of *a*(*t*) is governed by a second‐quantized Hamiltonian

(2) H=Δ+αVpump2αVpump222−βa^†a^a^†a^+αV0Vpump2a^†a^†+a^a^

where $\hat{a}$ and ${\hat{a}}^{†}$ are, respectively, the annihilation and creation operators for the topological Dirac‐vortex phonons, *β* is the Kerr nonlinear coefficient, and *α* is the electromechanical tuning coefficient (see Section S3 in the Supporting Information). The first term in Equation (2) represents the Dirac‐vortex mode whose frequency is shifted by the external electric field and the third‐order nonlinearity, while the second term represents the single‐mode squeezed interaction. The measured vibration quadrature in Figure 1f shows that the parametric pump *V*_pump_cos(2Ω*t*) can squeeze the Dirac‐vortex state (Figure S4, Supporting Information) and provide phase‐sensitive amplification (Figure S6, Supporting Information).

When the parametric amplification is sufficiently large to overcome the dissipation, the squeezed interaction can drive the zero solution *a*(*t*) = 0 of the system into instability. Consequently, any fluctuations can cause exponential growth of *a*(*t*) and finally the parametric phonon oscillation. The steady‐state power of the parametric oscillator is determined by the frequency detuning Δ and pump voltage *V*_pump_

**Figure 2:**
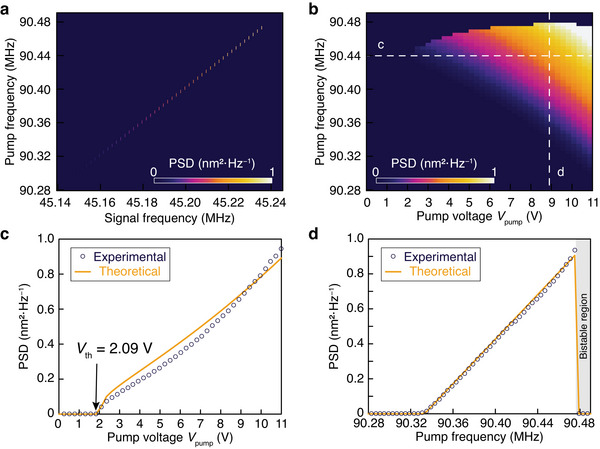
Experimental demonstration of parametric phonon oscillation from a Dirac‐vortex state under a coherent pump. a) Measured mechanical power spectral density (PSD) of the device with *R*_0_/*l*_0_ = 0.5 under the pump voltage *V*_pump_ = 11 V, showing that the oscillation frequency is always half of the pump frequency. b) Measured peak PSD of the device with *R*_0_/*l*_0_ = 0.5 as a function of the pump frequency and pump voltage *V*_pump_. c) Peak PSD as a function of *V*_pump_ with the pump frequency fixed at 90.44 MHz [along the white dashed line c in panel (b)]. d) Peak PSD as a function of the pump frequency with the pump voltage *V*_pump_ = 8.9 V [along the white dashed line d in panel (b)]. The bistable region is marked in gray. In panels (c) and (d), the dark blue circles and orange solid lines represent the measured data and theoretical fits, respectively.

(3) a(t)2=Δ+αVpump2αVpump222+αV0Vpump2−Vth2β

where *V*_th_ = *γ*/*αV*_0_ is the minimal threshold pump voltage when the frequency detuning satisfies $\Delta \;=\;-\alpha V_{\mathrm{th}}^{2}/2$ (see Section S4 in the Supporting Information). We characterized the phonon oscillation behavior of the device with *R*_0_/*l*_0_ = 0.5 by applying a coherent parametric pump. **Figure** **2**a plots the measured power spectral density (PSD) of phonon oscillation with *V*_pump_ = 11 V, which shows that the peak frequencies of the phonon oscillation spectra are always at Ω, i.e., half of the pump frequency 2Ω. Figure 2b plots the measured peak PSD as a function of the pump frequency 2Ω and pump voltage *V*_pump_, which agrees well with the theoretical prediction of Equation (3) (see Figure S5 and Section S4 in the Supporting Information). Figure 2c plots the peak PSD as a function of the pump voltage *V*_pump_ at a fixed pump frequency 2Ω/2π = 90.44 MHz. Figure 2d plots the peak PSD as a function of the pump frequency at a fixed pump voltage *V*_pump_ = 8.9 V, which shows a linear relationship between |*a*(*t*)|^2^ and the pump frequency 2Ω. By fitting the experimental results in Figure 2c,d with the analytical solution of Equation (3), we obtained the minimal threshold pump voltage *V*_th_ = 2.09 V, the Kerr nonlinear coefficient *β* = 0.392 kHz nm^−2^, and the electromechanical tuning coefficient *α* = 0.40 kHz V^−2^. Note that for 2Ω/2π > 90.477 MHz, the system enters a bistable region (gray‐shaded region in Figure 2d), where the parametric oscillation cannot be obtained because the zero solution *a*(*t*) = 0 is stable (see Section S4 in the Supporting Information).

**Figure 3:**
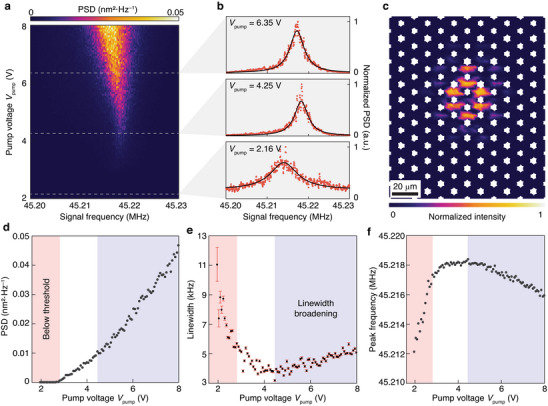
Experimental demonstration of parametric phonon oscillation from a Dirac‐vortex state under an incoherent white‐noise pump. a) Measured mechanical PSD of the device with *R*_0_/*l*_0_ = 0.5 under different pump voltages. b) Measured normalized PSD under pump voltages *V*_pump_ = 2.16, 4.25, and 6.35 V. The solid black lines are Lorentzian fits of the measured data. c) Measured intensity modal profile of the Dirac‐vortex state above the oscillation threshold. d) Measured peak PSD of the device as a function of *V*_pump_, showing a threshold pump voltage of 2.75 V. e) Fitted linewidth of the measured PSD as a function of *V*_pump_. The error bars represent standard deviation during the linewidth fitting. f) Measured peak frequency as a function of *V*_pump_. In panels (d)–(f), the region below the oscillation threshold is marked in pink, and the region of linewidth broadening is marked in light blue.

While the existence of a threshold in the stimulated phonon emission is a typical characteristic of lasing or oscillation, another fundamental characteristic of parametric oscillation is spectral linewidth narrowing above the threshold. To demonstrate that, we applied an incoherent white‐noise voltage centered at frequency 2Ω/2π = 90.43 MHz with a bandwidth of 20 kHz to parametrically pump the device. **Figure** **3**a,b shows the measured PSD under various pump voltage *V*_pump_. Figure 3c shows the measured modal profile above the oscillation threshold, which agrees well with the simulated result in Figure 1d. Figure 3d shows the measured peak PSD of the device as a function of *V*_pump_ under the incoherent pump, indicating a threshold pump voltage of 2.75 V. This threshold voltage is higher than that under a coherent pump (Figure 2c) because of a larger pump bandwidth. Figure 3e shows that the linewidth fitted from the measured spectra in Figure 3a reduces from 11.0 to 3.8 kHz when *V*_pump_ increases to 4.45 V, which is distinctive evidence of parametric oscillation. Figure 3f plots the fitted peak frequency of the phonon oscillation spectra as a function of the pump voltage. Below the threshold, the peak frequency increases rapidly, because the squeezed interaction changes the real part of the eigenfrequency of the Hamiltonian in Equation (2) before it is sufficiently strong to drive the system into instability (see Section S3 in the Supporting Information). On the other hand, when the pump voltage is sufficiently large, it induces a redshift to the oscillation frequency which is accompanied with slight broadening of the oscillation linewidth as shown in the light blue regions in Figure 3e,f.

**Figure 4:**
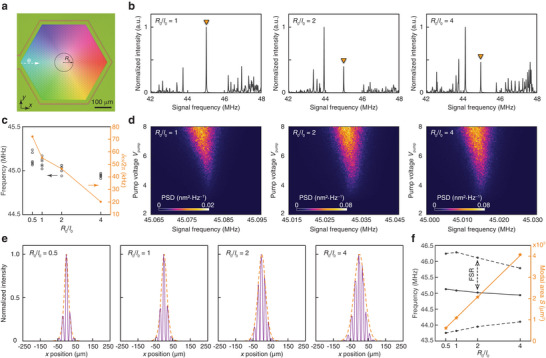
Experimental results of Dirac‐vortex parametric phonon oscillators with different modal areas. a) Optical microscope image of a fabricated device. The modal area *S* of the Dirac‐vortex state is controlled by the parameter *R*_0_. b) Measured mechanical intensity spectra of the devices with different *R*_0_ values, showing the existence of the Dirac‐vortex state in the bulk bandgap region of all these devices. c) Measured resonant frequencies of the Dirac‐vortex states with different *R*_0_/*l*_0_. The orange dots plot the frequency fluctuation *δω* measured from multiple devices with an identical design. We obtained *δω*/*ω*_0_ = 0.16%, 0.12%, 0.1%, and 0.045% for *R*_0_/*l*_0_ = 0.5, 1, 2, and 4, respectively. d) Measured mechanical PSD of the devices with *R*_0_/*l*_0_ = 1, 2, and 4 under an incoherent white‐noise parametric pump with a frequency bandwidth of 20 kHz. e) Measured oscillation intensity modal profiles |*f*_0_(**r**)|^2^ (purple solid lines) along the *x* direction as indicated by the white dashed arrow in panel (a) from the devices with different *R*_0_ values. The orange dashed lines plot the fitted envelope function |*g*_0_(|**r**|)|^2^. f) Measured resonant frequencies (purple dots) of the Dirac‐vortex state and its sidebands and the fitted modal area *S* (orange stars) of the Dirac‐vortex state from the devices with *R*_0_/*l*_0_ = 0.5, 1, 2, and 4.

We further investigated the Dirac‐vortex parametric phonon oscillators with different *R*_0_ (**Figure** **4**a). Figure 4b shows the experimental mechanical intensity spectra of the devices with different *R*_0_ measured under direct actuation at frequencies from 42 to 48 MHz, confirming the existence of the Dirac‐vortex states in the bulk bandgap region. Additionally, Figure 4c shows that the resonant frequencies of devices with an identical design can vary due to fabrication‐induced disorders. The relative frequency fluctuation *δω*/*ω*_0_ (*δω*: standard deviation of the resonant frequency) reduces from 0.16% to 0.045% as *R*_0_/*l*_0_ is increased from 0.5 to 4. Note that the frequency fluctuation due to fabrication‐induced disorders is considerably smaller than typical nontopological nanomechanical devices.^[^ ^33^ ^]^ These results also prove that the resonant frequency of Dirac‐vortex state is pinned to the frequency of the bulk Dirac point. Furthermore, we experimentally confirmed that all the devices with different *R*_0_ values can support coherent phonon oscillation under an incoherent parametric pump, as shown by the measured PSD in Figure 4d. Figure 4e shows the intensity modal profiles |*f*_0_(**r**)|^2^ measured above the oscillation threshold along the *x* direction as indicated by the white dashed arrow in Figure 4a. Their envelopes (orange dashed lines in Figure 4e) agree well with the analytic function ${|g_{0}\left(\left|r\right|\right)|}^{2}={\left[\cosh(\left|r\right|/R_{0})\right]}^{-R_{0}/\varsigma }$, where the experimentally fitted *ς* was found to be 0.52*l*_0_. Figure 4f plots the devices’ modal area $\;S=\pi D_{\mathrm{eff}}^{2}/4$ as a function of *R*_0_ (orange stars), where *D*_eff_ is the effective modal diameter defined by the full width at half maximum of the fitted function |*g*_0_(|**r**|)|^2^ in Figure 4e. Meanwhile, Figure 4f also shows the measured resonant frequencies of the desired Dirac‐vortex state and its sideband states (purple dots). These results confirm that the FSR of the Dirac‐vortex states defies the conventional inverse relationship FSR ∼ 1/*S*. These results also show that the frequency variation caused by fabrication disorders can be suppressed by increasing the cavity size while the free spectral range reduces at a much slower rate (also see Figure S7 in the Supporting Information), which indicates that the Dirac‐vortex cavities are suitable for realizing large‐area single‐mode lasers.

## 3 Conclusion

In conclusion, we experimentally demonstrated a topological Dirac‐vortex parametric phonon oscillator by harnessing the strong squeezed interaction in a nano‐electromechanical system. Our results will excite broad interests in the following aspects. First, our topological phononic system with strong squeezed interaction offers a practical experimental platform for investigating the interplay between topology and nonlinearity. It can be used for studying many important phenomena such as topological solitons, topological entangled phonon‐pair generation, topological nonclassical state preparation, and topological synchronization and chaos. Second, the experimentally realized parametric gain offers a promising strategy to investigate the non‐Hermitian topological physics, which is still experimentally elusive in the phononic domain. Third, considering that a similar nonlinear Hamiltonian has already been adopted for bit storage and flipping operations in nano‐electromechanical systems,^[^ ^34^ ^]^ our results can readily lead to topologically protected phononic computing technologies. Last, by carefully designing photonic crystals in active optical materials or adopting the design in materials having strong phonon–photon interactions,^[^ ^35^ ^]^ one can achieve large‐area single‐mode topological optical lasers or even phonon–polariton lasers.

## 4 Experimental Section

### Simulation

Commercial software (COMSOL Multiphysics) was used to simulate the mechanical Dirac‐vortex state with a finite‐element method. The simulation was done by using a 2D eigenfrequency analysis due to limit of computational resources. The following parameters were adopted for the silicon nitride membranes in the simulation: mass density = 3.1 × 10^3^ kg m^−3^, thickness = 140 nm, Young's modulus = 250 GPa, Poisson's ratio = 0.23, and isotropic residual stress = 1.15 GPa. The deposited 40‐nm‐thick aluminum layer was treated as a mass load to the silicon nitride membranes. The small etched holes in the silicon nitride membranes were ignored due to their negligible influence on the simulation results.

Bulk states and bulk band diagrams in 3D simulation were also calculated with substrate, supporting pillars, and the etched holes presented. It was shown that the supporting pillars and the etched holes would only slightly shift the band's frequency of the phononic crystal but would not influence the topological properties, because the height of the supporting pillars and the diameter of the holes are orders of magnitude smaller than the lattice constant. The 3D simulation agreed well with the 2D simulation with the configurations described above.

### Fabrication

The Dirac‐vortex cavities were fabricated on a silicon‐nitride‐on‐insulator wafer, which had a 150‐nm silicon nitride layer on 180‐nm buried oxide on a heavily doped silicon substrate. The fabrication processes were as follows (Figure S1, Supporting Information): i) defining the patterns of the small holes and the electrode windows in an electron‐beam resist by electron‐beam lithography; ii) transferring the patterns in the electron‐beam resist to the silicon nitride layer by plasma dry etching; iii) using a buried‐oxide etchant to etch away the oxide in proximity of the small holes created in the silicon nitride layer in step (ii)—this wet‐etching process resulted in an undercut depth of ≈2.9 µm as well as reduction of the silicon nitride thickness from 150 to 140 nm; iv) drying the devices in a critical‐point dryer; and v) depositing 40‐nm aluminum on the devices by electron‐beam evaporation.

### Measurement

The same measurement platform was used as that in ref. [27]. During the measurement, the nano‐electromechanical devices were placed inside a vacuum chamber with a pressure of ≈4.5 × 10^−3^ mBar. An a.c. pump voltage was combined with a d.c. bias voltage (*V*_0_ = 10 V) to actuate the mechanical motions of the Dirac‐vortex cavity. A homebuilt optical Michelson interferometer was employed to measure the mechanical motions of the devices, the signal of which was collected by a high‐speed photodetector. In Figures 1e and 4b,c, the devices were directly actuated and their mechanical intensity spectra were measured with a network analyzer by sweeping the frequency near the resonant frequency *ω*_0_ (Figure S2a, Supporting Information). For the quadrature measurement (Figure 1f; Figure S4, Supporting Information), a coherent pump *V*_pump_ (frequency 2Ω = 2*ω*_0_) was used to squeeze the mechanical state and a weak white‐noise signal (center frequency *ω*_0_, bandwidth = 50 kHz) was injected to directly actuate the mechanical state. The measured signal was sent to a two‐phase lock‐in amplifier to analyze the quadrature of the mechanical state. For the parametric amplification measurement (Figure S6, Supporting Information), a coherent pump *V*_pump_ (frequency 2Ω = 2*ω*_0_) was used to provide parametric amplification, and a network analyzer was used to drive the device at frequency *ω*_0_ under very low power (−40 dBm) and analyze the modified mechanical response under the parametric pump *V*_pump_. For the parametric phonon oscillation measurement, a signal generator was used to produce a coherent (Figure 2) or an incoherent (Figures 3 and 4d,e) parametric pump, and the mechanical PSD was analyzed by using an electrical spectrum analyzer with a measurement resolution bandwidth of 200 Hz (Figure S2b, Supporting Information).

## Conflict of Interest

The authors declare no conflict of interest.

## Supporting information

Supporting Information

## Data Availability

The data that support the findings of this study are available from the corresponding author upon reasonable request.
