# Supplementary material for: A Topological Parametric Phonon Oscillator
Source: Adv Mater. 2024 Oct 15;37(2):2309015. [Article in Italian] doi: 10.1002/adma.202309015 (PMC11733721; doi:10.1002/adma.202309015)
Supplement: Supplementary file 1 — Supporting Information [file ADMA-37-2309015-s001.pdf]

ADVANCED MATERIALS

Supporting Information

for *Adv. Mater.*, DOI 10.1002/adma.202309015

A Topological Parametric Phonon Oscillator

*Xiang Xi, Jingwen Ma and Xiankai Sun**

Supporting Information for "A topological parametric phonon oscillator"

Xiang Xi†, Jingwen Ma†, and Xiankai Sun*

Department of Electronic Engineering, The Chinese University of Hong Kong, Shatin, New Territories, Hong Kong

†*These authors contributed equally to this work*

**Corresponding author: xksun@cuhk.edu.hk*

Contents

1. **Device fabrication and measurement methods** 2
    - **1.1. Device fabrication** 2
    - **1.2. Device measurement** 2
2. **Theoretical analysis of nanomechanical Dirac-vortex states** 2
    - **2.1. Effective bulk Hamiltonian of the nanomechanical topological crystal** 3
    - **2.2. Analytical solution of the Dirac-vortex states without external electrical pump** 5
3. **Squeezed Dirac-vortex states under external electrical pump** 7
4. **Parametric oscillation behavior of the Dirac-vortex states** 9
5. **Topological parametric amplification of mechanical signals** 11
6. **Additional data** 13

1. Device fabrication and measurement methods

1.1. Device fabrication

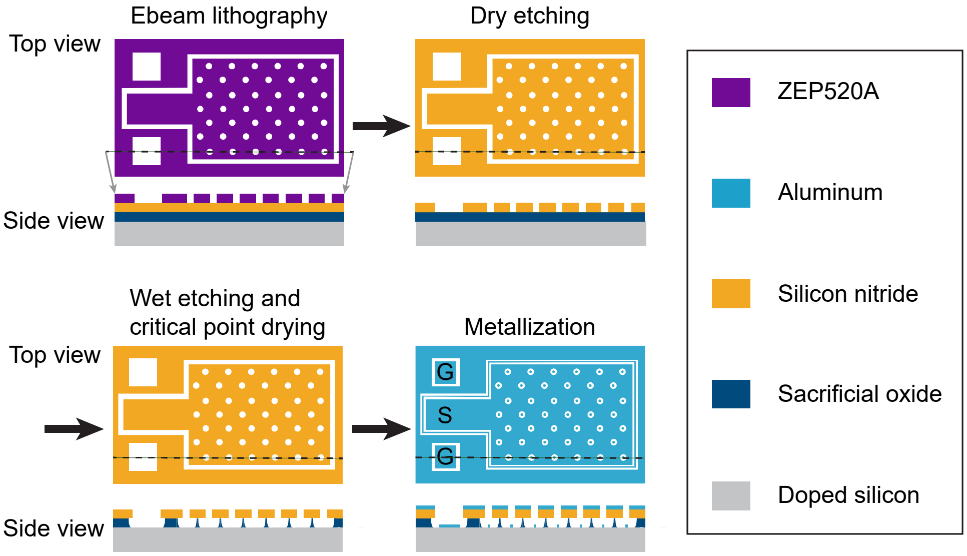

Figure S1. Fabrication process flowchart of the devices. G, ground electrode; S, signal electrode.

1.2. Device measurement

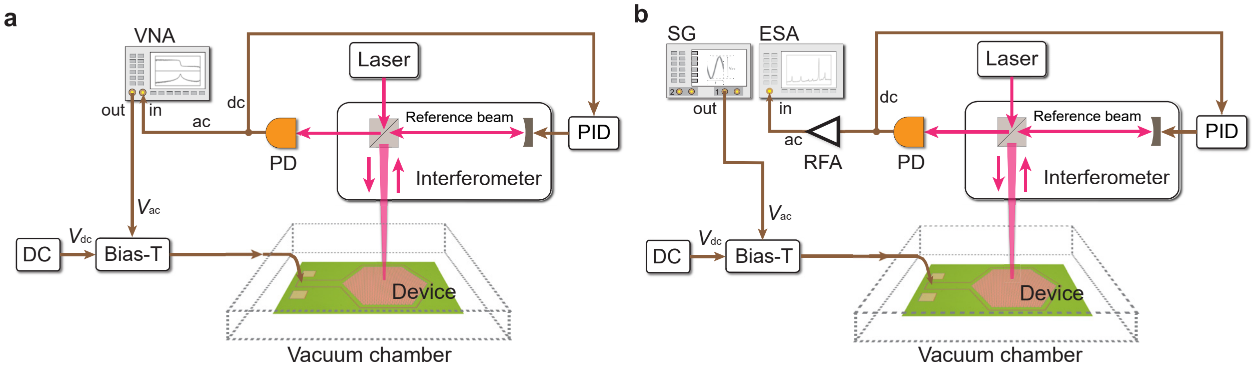

Figure S2. Measurement setup. **a**, Experimental setup for direct measurement of the mechanical intensity spectra. Bias-T, bias tee; DC, d.c. voltage source; PD, photodetector; PID, proportional–integral–derivative controller; VNA, vector network analyzer. **b**, Experimental setup for measurement of parametric phonon oscillation. ESA, electrical spectrum analyzer; RFA, radiofrequency amplifier; SG, signal generator.

2. Theoretical analysis of nanomechanical Dirac-vortex states

The elastic waves in the 2D nanomechanical crystal are governed by the equation

$$\rho h \frac{\partial^2 W(\mathbf{r},t)}{\partial t^2} + D\nabla^4 W(\mathbf{r},t)\cdot\zeta(\mathbf{r}) = \frac{\varepsilon V^2}{2\left[d - W(\mathbf{r},t)\right]^2}, \tag{S1}$$

where ρ, h, and $D = Eh^3/[12(1-\nu^2)]$ are respectively the density, thickness, and bending stiffness of the suspended silicon nitride membranes. $W(\mathbf{r}, t)$ is the elastic displacement field in the z direction. ε is the effective permittivity, V and d are respectively the applied voltage and distance between the electrode and the ground. The function $\zeta(\mathbf{r})$ in Eq. (S1) defines the geometry of the suspended silicon nitride membranes

$$\zeta(\mathbf{r}) = \begin{cases} 1, & \mathbf{r}\in A_{\text{suspended}}, \\ 0, & \mathbf{r}\in A_{\text{unsuspended}}. \end{cases}$$

2.1. Effective bulk Hamiltonian of the nanomechanical topological crystal

We first analyze the effective bulk Hamiltonian of the nanomechanical topological crystal by setting the right-hand-side term of Eq. (S1) to be zero and assuming that the geometry function $\zeta(\mathbf{r})$ is strictly periodic. Assuming that Eq. (S1) has a solution $W_{\mathbf{k}}(\mathbf{r}, t) = f_{\mathbf{k}}(\mathbf{r})e^{j\omega_{\mathbf{k}}t}$ + h.c., the complex modal profile $f_{\mathbf{k}}(\mathbf{r})$ satisfies

$$-\rho h\omega_{\mathbf{k}}^2 f_{\mathbf{k}}(\mathbf{r}) + D\nabla^4 f_{\mathbf{k}}(\mathbf{r})\cdot\zeta(\mathbf{r}) = 0. \tag{S2}$$

Here $f_{\mathbf{k}}(\mathbf{r})$ can be decomposed as

$$f_{\mathbf{k}}(\mathbf{r}) = \sum_{\mathbf{G}} c_{\mathbf{k},\mathbf{G}} \exp(j\mathbf{k}\cdot\mathbf{r} + j\mathbf{G}\cdot\mathbf{r}), \tag{S3}$$

where $\mathbf{G}$ represents the reciprocal vectors of the crystal. Here, we take six reciprocal vectors $\mathbf{G}_m$ (m = 1–6) into consideration. Substituting Eq. (S3) into Eq. (S2) leads to an eigenvalue problem

$$\omega_{\mathbf{k}}^2 c_{\mathbf{G}'} = \frac{D}{\rho h}\sum_{\mathbf{G}} c_{\mathbf{k},\mathbf{G}} \left|\mathbf{k}+\mathbf{G}\right|^4 \cdot \eta(\mathbf{G}-\mathbf{G}') \approx \frac{D}{\rho h}\sum_{\mathbf{G}} c_{\mathbf{k},\mathbf{G}}\left[\left|\mathbf{G}\right|^4 + 4\left|\mathbf{G}\right|^2(\mathbf{k}\cdot\mathbf{G})\right]\cdot\eta(\mathbf{G}-\mathbf{G}').$$

Here $\eta(\mathbf{k})$ is the Fourier transform of the geometry function $\zeta(\mathbf{r})$:

$$\eta(\mathbf{k}) = \int_{\text{unit cell}} \zeta(\mathbf{r})\exp(j\mathbf{k}\cdot\mathbf{r})\cdot d\mathbf{r}.$$

We will find out the values of $\eta(\mathbf{k})$ at the $\mathbf{\Gamma}$, $\mathbf{G}_m$, and $\mathbf{P}_m$ points (m = 1–6), which are shown in Fig. S3. Specifically, the geometric parameter $\delta_t = \delta_0\sin\theta$ leads to a nonzero $\eta(\mathbf{G}_m) = \alpha_t$ (m = 1–6), and the geometric parameter $\delta_i = \delta_0\cos\theta$ leads to a nonzero imaginary part of $\eta(\mathbf{P}_m)$ such that $\eta(\mathbf{P}_m) = \eta_1 + j\cdot\alpha_i$ for m = 1–3 and $\eta(\mathbf{P}_m) = \eta_1 - j\cdot\alpha_i$ for m = 4–6. Here, α_t and α_i are proportional to the geometric parameter δ_t and δ_i, respectively.

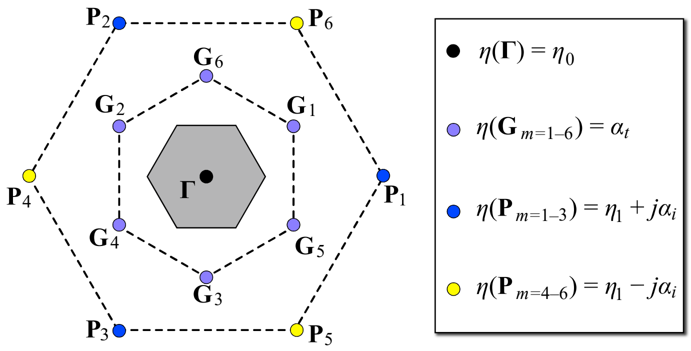

Figure S3. Theoretical analysis of the nanomechanical crystal using a plane-wave expansion method. The gray shaded hexagon indicates the first Brillouin zone of the nanomechanical crystal.

Considering the specific values of $\eta(\mathbf{k})$ at the $\boldsymbol{\Gamma}$, $\mathbf{G}_m$, and $\mathbf{P}_m$ points (m = 1–6) shown in Fig. S3, we obtain

$$\lambda_{\mathbf{k}}\mathbf{c}_{\mathbf{k}} = \mathbf{H}_{\mathbf{k}}\mathbf{c}_{\mathbf{k}}, \tag{S4}$$

with the eigenstate vector $\mathbf{c}_{\mathbf{k}} = (c_{\mathbf{k},\mathbf{G}_{1,+}}, c_{\mathbf{k},\mathbf{G}_{2,+}}, c_{\mathbf{k},\mathbf{G}_{3,+}}, c_{\mathbf{k},\mathbf{G}_{1,-}}, c_{\mathbf{k},\mathbf{G}_{2,-}}, c_{\mathbf{k},\mathbf{G}_{3,-}})^{\mathrm{T}}$, the eigenvalue $\lambda_{\mathbf{k}} = \omega_{\mathbf{k}}^2 \rho h/D|G|^4 - (\eta_0 - \eta_1)$, and the Hamiltonian

$$\mathbf{H}_{\mathbf{k}} = \left(\eta_0 \cdot \mathbf{I}_3 \otimes \boldsymbol{\sigma}_0 + \eta_1 \cdot \mathbf{M}_1 \otimes \boldsymbol{\sigma}_0 + \alpha_t \cdot \mathbf{M}_1 \otimes \boldsymbol{\sigma}_x + j\alpha_i \cdot \mathbf{M}_2 \otimes \boldsymbol{\sigma}_z\right) \cdot \left(\mathbf{I}_3 \otimes \boldsymbol{\sigma}_0 + \frac{4}{G^2}\mathbf{M}_3 \otimes \boldsymbol{\sigma}_z\right) - (\eta_0 - \eta_1) \cdot \mathbf{I}_3 \otimes \boldsymbol{\sigma}_0.$$

In the above equations, the symbols are defined as $G = |\mathbf{G}_m|$ (m = 1–3),

$$\boldsymbol{\sigma}_0 = \begin{bmatrix} 1 & 0 \\ 0 & 1 \end{bmatrix}, \boldsymbol{\sigma}_x = \begin{bmatrix} 0 & 1 \\ 1 & 0 \end{bmatrix}, \boldsymbol{\sigma}_z = \begin{bmatrix} 1 & 0 \\ 0 & -1 \end{bmatrix},$$

$$\mathbf{I}_3 = \begin{bmatrix} 1 & 0 & 0 \\ 0 & 1 & 0 \\ 0 & 0 & 1 \end{bmatrix}, \mathbf{M}_1 = \begin{bmatrix} 0 & 1 & 1 \\ 1 & 0 & 1 \\ 1 & 1 & 0 \end{bmatrix}, \mathbf{M}_2 = \begin{bmatrix} 0 & 1 & -1 \\ -1 & 0 & 1 \\ 1 & -1 & 0 \end{bmatrix}, \mathbf{M}_3 = \begin{bmatrix} \mathbf{k}\cdot\mathbf{G}_1 & 0 & 0 \\ 0 & \mathbf{k}\cdot\mathbf{G}_2 & 0 \\ 0 & 0 & \mathbf{k}\cdot\mathbf{G}_3 \end{bmatrix}.$$

The Hamiltonian $\mathbf{H}_{\mathbf{k}}$ in Eq. (S4) has eigenvalues

$$(\lambda_{1,\uparrow}, \lambda_{2,\uparrow}, \lambda_{1,\downarrow}, \lambda_{2,\downarrow}) = (-\Delta_0/2, \Delta_0/2, -\Delta_0/2, \Delta_0/2)$$

at the $\boldsymbol{\Gamma}$ point ($\mathbf{k}$ = 0). As the opened bandgap $\Delta_0 = 2\sqrt{3\alpha_i^2 + \alpha_t^2}$ is proportional to the geometric parameter $\delta_0 = \sqrt{\delta_i^2 + \delta_t^2}$ and does not depend on $\tan\theta = \delta_t/\delta_i$, the parameters α_i and α_t can be expressed as $(\sqrt{3}\alpha_i, \alpha_t) = \Delta_0 \cdot (\cos\theta, \sin\theta)/2$. Note that the Hamiltonian in Eq. (S4) has six eigenvalues in total, but we consider four of them, because the other two have eigenvalues far away from $\lambda_{n,\uparrow\downarrow}$ (n = 1, 2).

To further investigate the underlying physics of the nanomechanical crystal, we define another four states which are superpositions of the plane waves with wave vectors $\mathbf{G}_m$ (m = 1–6)

$$\begin{cases} \left|\Psi_{\pm,\downarrow}\right\rangle = \sum_{m=1}^{3} \exp\left(\frac{-j2\pi m}{3}\right)\cdot\left[\exp(-j\mathbf{G}_m\cdot\mathbf{r}) \pm j\cdot\exp(j\mathbf{G}_m\cdot\mathbf{r})\right], \\ \left|\Psi_{\pm,\uparrow}\right\rangle = \sum_{m=1}^{3} \exp\left(\frac{j2\pi m}{3}\right)\cdot\left[\pm\exp(j\mathbf{G}_m\cdot\mathbf{r}) + j\cdot\exp(-j\mathbf{G}_m\cdot\mathbf{r})\right], \end{cases} \tag{S5}$$

so that $f_{\mathbf{k}}(\mathbf{r})$ can be decomposed as

$$f_{\mathbf{k}}(\mathbf{r}) = \sum_{n,s} \exp(j\mathbf{k}\cdot\mathbf{r})\cdot c_{\mathbf{k},n,s}\left|\psi_{n,s}\right\rangle, \quad (n=\pm, s=\downarrow\uparrow). \tag{S6}$$

With the states $\left(\left|\psi_{+,\downarrow}\right\rangle, \left|\psi_{+,\uparrow}\right\rangle, \left|\psi_{-,\downarrow}\right\rangle, \left|\psi_{-,\uparrow}\right\rangle\right)$ as the basis, Eq. (S4) can be reduced to

$$\lambda_{\mathbf{k}}\mathbf{c}_{\mathbf{k}} = \mathbf{H}(\mathbf{k})\cdot\mathbf{c}_{\mathbf{k}}, \tag{S7}$$

with the eigenstate vector $\mathbf{c}_{\mathbf{k}} = (c_{\mathbf{k},+,\downarrow}, c_{\mathbf{k},+,\uparrow}, c_{\mathbf{k},-,\downarrow}, c_{\mathbf{k},-,\uparrow})^{\mathrm{T}}$ and the Hamiltonian

$$\mathbf{H}(\mathbf{k}) = v_D\cdot(\boldsymbol{\sigma}_x k_x + \boldsymbol{\sigma}_y k_y) - \frac{\Delta_0}{2}\boldsymbol{\sigma}_z(\boldsymbol{\tau}_x\cos\theta + \boldsymbol{\tau}_y\sin\theta), \tag{S8}$$

where $\boldsymbol{\sigma}_x$, $\boldsymbol{\sigma}_y$, $\boldsymbol{\sigma}_z$, and $\boldsymbol{\tau}_z$ are the Pauli matrices, and $v_D = 2\left(\eta_1 - \eta_0\right)/G$ is the effective Fermi velocity near the $\boldsymbol{\Gamma}$ point. In Eq. (S8), the first term indicates the double Dirac-cone dispersion in the momentum space, and the second term indicates the effective masses that can gap the double Dirac cones. The Hamiltonian **H**(**k**) in Eq. (S8) is mathematically identical to the Jackiw–Rossi model, where $\left|\psi_{\pm,\downarrow\uparrow}\right\rangle$ represents charge-conjugate (+/−) Dirac fermions with opposite spins (↓/↑). Besides, similar to the Jackiw–Rossi model, the states $\left|\psi_{\pm,\downarrow\uparrow}\right\rangle$ in Eq. **Error! Reference source not found.** naturally satisfy the charge-conjugation symmetry

$$\begin{pmatrix} \left|\psi_{-,\downarrow}\right\rangle \\ \left|\psi_{-,\uparrow}\right\rangle \end{pmatrix} = -j\begin{pmatrix} \left|\psi_{+,\downarrow}\right\rangle \\ \left|\psi_{+,\uparrow}\right\rangle \end{pmatrix}^{\dagger}\boldsymbol{\sigma}_y. \tag{S9}$$

2.2. Analytical solution of the Dirac-vortex states without external electrical pump

In the previous discussion, we only focus on the geometry function $\zeta(\mathbf{r})$ that is strictly periodic with constant parameters Δ_0 and θ. We will consider a different case where the geometric parameters Δ_0 and θ are functions of the spatial position **r**. Assuming that Eq. (S1) has a solution taking the amplitude distribution of the nanomechanical Dirac-vortex state $W_0(\mathbf{r}, t) = f_0(\mathbf{r})e^{j\omega_{\mathrm{int}}t} + \mathrm{h.c.}$ with ω_{int} describing the intrinsic resonant frequency of the Dirac-vortex state, the modal profile $f_0(\mathbf{r})$ satisfies

$$-\rho h\omega_{\text{int}}^{2} f_0(\mathbf{r}) + D\nabla^4 f_0(\mathbf{r})\cdot\zeta(\mathbf{r}) = 0, \tag{S10}$$

with

$$f_0(\mathbf{r}) = \sum_{n,s} c_{n,s}(\mathbf{r})\left|\psi_{n,s}\right\rangle, \quad (n=\pm, s=\downarrow\uparrow). \tag{S11}$$

Similar to Eqs. (S7) and (S8), the Dirac-vortex state is governed by

$$\lambda_0\mathbf{c}(\mathbf{r}) = \mathbf{H}(\mathbf{r})\cdot\mathbf{c}(\mathbf{r}), \tag{S12}$$

with the spatially dependent vector $\mathbf{c}(\mathbf{r}) = [c_{+,\downarrow}(\mathbf{r}), c_{+,\uparrow}(\mathbf{r}), c_{-,\downarrow}(\mathbf{r}), c_{-,\uparrow}(\mathbf{r})]^{\mathrm{T}}$, the eigenvalue $\lambda_0 = \omega_0^2\rho h/D|G|^4 - (\eta_0-\eta_1)$, and the real-space Hamiltonian

$$\mathbf{H}(\mathbf{r}) = -jv_D\cdot(\boldsymbol{\sigma}_x\partial_x + \boldsymbol{\sigma}_y\partial_y) - \frac{\Delta_0}{2}\boldsymbol{\sigma}_z(\boldsymbol{\tau}_x\cos\theta + \boldsymbol{\tau}_y\sin\theta). \tag{S13}$$

With polar coordinates $\mathbf{r} = R\cdot(\cos\varphi, \sin\varphi)$, we focus on the zero mode with $\lambda_0 = 0$, so that Eqs. (S12) and (S13) lead to the following equations

$$\begin{cases} -jv_{\mathrm{D}}e^{-j\varphi}\left(\partial_R - \dfrac{j}{R}\partial_\varphi\right)c_{+,\uparrow}(\mathbf{r}) - \dfrac{\Delta_0(\mathbf{r})e^{-j\theta(\mathbf{r})}}{2}c_{+,\uparrow}^{*}(\mathbf{r}) = 0, \\ -jv_{\mathrm{D}}e^{j\varphi}\left(\partial_R + \dfrac{j}{R}\partial_\varphi\right)c_{+,\downarrow}(\mathbf{r}) - \dfrac{\Delta_0(\mathbf{r})e^{-j\theta(\mathbf{r})}}{2}c_{+,\downarrow}^{*}(\mathbf{r}) = 0. \end{cases} \tag{S14}$$

Note that the values of $c_{-,\downarrow\uparrow}(\mathbf{r})$ can be determined by the relationship $c_{-,\downarrow}(\mathbf{r}) = c_{+,\uparrow}^{*}(\mathbf{r})$ and $c_{-,\uparrow}(\mathbf{r}) = -c_{+,\downarrow}^{*}(\mathbf{r})$. We focus on a special case with $\Delta_0(R) = \Delta_{\max}\cdot\tanh(R/R_0)$ and $\theta(\varphi) = w\cdot\varphi + \theta_0$, where R_0 controls the size of the cavity, $w = 1$ is the winding number of the vortex, and θ_0 is the value of $\theta(\varphi)$ at $\varphi = 0$. We assume that the solution of Eq. (S14) is $c_{+,\downarrow\uparrow}(\mathbf{r}) = g_{\downarrow\uparrow}(R)\cdot\exp\left(jp_{\downarrow\uparrow}\varphi + j\vartheta_{\downarrow\uparrow}\right)$, where $g_{\downarrow\uparrow}(R)$ is the amplitude distribution along the radial direction, the integer $p_{\downarrow\uparrow}$ is the angular quantum number, and $\vartheta_{\downarrow\uparrow}$ is the additional phase term of $c_{+,\downarrow\uparrow}(\mathbf{r})$, then Eq. (S14) can be rewritten as

$$\begin{cases} jv_{\mathrm{D}}\left(\partial_R + \dfrac{p_\uparrow}{R}\right)g_\uparrow(R) + \dfrac{\Delta_0(R)}{2}g_\uparrow(R)e^{-2j(p_\uparrow\varphi+\vartheta_\uparrow+\theta_0/2)} = 0, \\ jv_{\mathrm{D}}\left(\partial_R - \dfrac{p_\downarrow}{R}\right)g_\downarrow(R) + \dfrac{\Delta_0(R)}{2}g_\downarrow(R)e^{-2j(p_\downarrow\varphi+\varphi+\vartheta_\downarrow+\theta_0/2)} = 0. \end{cases} \tag{S15}$$

As Eq. (S15) is valid for arbitrary φ values, we obtain $p_\uparrow = 0$ and $p_\downarrow = -1$. Besides, as $g_{\downarrow\uparrow}(R)$ is always real, we obtain $\vartheta_{\uparrow\downarrow} = -\theta_0/2 \pm \pi/4$, so that Eq. (S15) can be reduced to

$$\begin{cases} \partial_R g_\uparrow(R) = \dfrac{\Delta_0(R)}{2v_\mathrm{D}} g_\uparrow(R) & \left(\vartheta_\uparrow = -\theta_0/2 + \dfrac{\pi}{4}\right), \\ \partial_R g_\uparrow(R) = -\dfrac{\Delta_0(R)}{2v_\mathrm{D}} g_\uparrow(R) & \left(\vartheta_\uparrow = -\theta_0/2 - \dfrac{\pi}{4}\right), \end{cases} \quad \text{(S16)}$$

and

$$\begin{cases} \partial_R g_\downarrow(R) = \left(-\dfrac{1}{R} + \dfrac{\Delta_0(R)}{2v_\mathrm{D}}\right) g_\downarrow(R) & \left(\vartheta_\downarrow = -\theta_0/2 + \dfrac{\pi}{4}\right), \\ \partial_R g_\downarrow(R) = \left(-\dfrac{1}{R} - \dfrac{\Delta_0(R)}{2v_\mathrm{D}}\right) g_\downarrow(R) & \left(\vartheta_\downarrow = -\theta_0/2 - \dfrac{\pi}{4}\right). \end{cases} \quad \text{(S17)}$$

Considering the boundary condition that $g_{\downarrow\uparrow}(R = 0)$ is finite and $g_{\downarrow\uparrow}(R = +\infty)$ is zero, we find that Eq. (S16) has a nonzero solution

$$g_\uparrow(R) = \exp\left[-\int_0^R \frac{\Delta_\mathrm{max} \tanh(r/R_0)}{2v_\mathrm{D}} dr\right] = \left[\cosh\left(R/R_0\right)\right]^{\frac{-\Delta_\mathrm{max} R_0}{2v_\mathrm{D}}} \quad \text{(S18)}$$

only when $\vartheta_\uparrow = -\theta_0/2 - \pi/4$, while Eq. (S17) always has zero solutions $g_\downarrow(R) = 0$.

In conclusion, the modal profile of the Dirac-vortex state with parameters $\Delta(R) = \Delta_\mathrm{max}\cdot\tanh(R/R_0)$ and $\theta(\varphi) = \varphi$ is

$$f_0(\mathbf{r}) = g_0(R)\cdot\left|\psi_0\right\rangle = \left[\cosh(R/R_0)\right]^{-R_0/\varsigma}\cdot\left(e^{-j(\theta_0/2+\pi/4)}\left|\psi_{+,\uparrow}\right\rangle + e^{j(\theta_0/2+\pi/4)}\left|\psi_{-,\downarrow}\right\rangle\right), \quad \text{(S19)}$$

with $\cosh(x) = \frac{\exp(x)+\exp(-x)}{2}$ and $\varsigma = \frac{2v_\mathrm{D}}{\Delta_\mathrm{max}}$. Note that $f_0(\mathbf{r})$ naturally satisfies $f_0(\mathbf{r}) = f_0^*(\mathbf{r})$. Here, we only consider $\theta(\varphi) = w\cdot\varphi + \theta_0$ with $w = 1$. The general solution of the Dirac-vortex mode with arbitrary w values can be found in Ref. S3. From Eq. (S19) one can also find that the Dirac-vortex mode exhibits an interesting property: adiabatic variation of θ_0 from 0 to 2π produces a nontrivial geometric phase π. This phenomenon is closely related to the braiding of the Majorana modes.

3. Squeezed Dirac-vortex states under external electrical pump

In this section, we analyze the dynamics of nanomechanical Dirac-vortex states under an external drive. In our experiment, the applied voltage is $V = V_0 + V_\mathrm{pump}\cos(2\Omega t)$, which is a combination of a d.c. voltage V_0 and an a.c. pump voltage with amplitude V_pump and frequency 2Ω. Here Ω is set near the intrinsic resonant frequency of

the Dirac-vortex state. Considering that $W(\mathbf{r}, t)$ is far less than the distance d between the electrode and the ground, Eq. (S1) can be rewritten as

$$\rho h\frac{\partial^2 W(\mathbf{r},t)}{\partial t^2}+D\nabla^4 W(\mathbf{r},t)\cdot\zeta(\mathbf{r})=\frac{\varepsilon\left(V_0+V_{\text{pump}}\cos(2\Omega t)\right)^2}{2d^2}+\frac{\varepsilon\left(V_0+V_{\text{pump}}\cos(2\Omega t)\right)^2}{d^3}W(\mathbf{r},t).$$

We assume $W(\mathbf{r}, t) = a(t)f_0(\mathbf{r})e^{j\Omega t}$ + h.c., where $a(t)$ describes the temporal dynamics of the Dirac-vortex state. Retaining the terms containing $e^{j\Omega t}$, we obtain

$$\rho h\left\{2j\Omega\frac{\partial a(t)}{\partial t}-\left[(\Omega-\omega_{\text{int}})^2+2\omega_0(\Omega-\omega_{\text{int}})+\frac{\varepsilon V_0^2}{\rho hd^3}+\frac{V_{\text{pump}}^2\varepsilon}{2\rho hd^3}\right]a(t)-\frac{\varepsilon V_0 V_{\text{pump}}a^*(t)}{\rho hd^3}\right\}f_0(\mathbf{r})$$
$$+\left[-\rho h\omega_0^2 f_0(\mathbf{r})+D\nabla^4 f_0(\mathbf{r})\right]a(t)=0.$$

Considering that $\Omega-\omega_{\text{int}} << \omega_{\text{int}}$ and $f_0(\mathbf{r})$ naturally satisfies Eq. (S10), we obtain

$$j\frac{\partial a(t)}{\partial t}=\left(\Delta+\alpha V_{\text{pump}}^2/2\right)a(t)+\alpha V_0 V_{\text{pump}}a^*(t), \tag{S20}$$

with the electromechanical tuning coefficient $\alpha=\frac{\varepsilon}{2\rho hd^3\omega_0}$, the relevant frequency detuning $\Delta = \Omega - \omega_0$. Here $\omega_0=\omega_{\text{int}}-\alpha V_0^2$ is the dressed resonant frequency of the Dirac-vortex state after considering the electromechanical tuning effect of the d.c. voltage V_0. Equation (S20) corresponds to a Hamiltonian

$$H=\left(\Delta+\alpha V_{\text{pump}}^2/2\right)\hat{a}^\dagger\hat{a}+\frac{\alpha V_0 V_{\text{pump}}}{2}\left(\hat{a}^\dagger\hat{a}^\dagger+\hat{a}\hat{a}\right), \tag{S21}$$

where $\hat{a}$ and $\hat{a}^\dagger$ represent the annihilation and creation operator, respectively. According to Eq. (S21), the a.c. pump field induces the particle-hole squeezing interaction that breaks the conservation of particle number of the system. Figure S4 shows the measured squeezed quadrature of the nanoelectromechanical Dirac-vortex states.

To gain further insight into the squeezing term, we conduct Bogoliubov transformation so that the Hamiltonian in Eq. (S21) can be rewritten as

$$H=j\sqrt{\left(\alpha V_0 V_{\text{pump}}\right)^2-\left(\Delta+\alpha V_{\text{pump}}^2/2\right)^2}\,\hat{b}^\dagger\hat{b}, \tag{S22}$$

with

$$\hat{b}=\frac{u+u^{-1}}{2}\hat{a}+\frac{u-u^{-1}}{2}\hat{a}^\dagger \tag{S23}$$

and

$$u=\left(\frac{\alpha V_0 V_{\text{pump}}+\Delta+\alpha V_{\text{pump}}^2/2}{\alpha V_0 V_{\text{pump}}-\Delta-\alpha V_{\text{pump}}^2/2}\right)^{1/4}\cdot e^{j\pi/4}.$$

According to Eq. (S22), the dressed mode $\hat{b}$ experiences an additional gain from the squeezing interaction when $\alpha V_0 V_{\text{pump}} > |\Delta+\alpha V_{\text{pump}}^2/2|$. When this squeezing-induced gain is higher than the intrinsic dissipation rate of the Dirac-vortex mode, the system is parametrically driven into an instable regime, leading to parametric phonon oscillation. Note that the dressed mode $\hat{b}$ has a real eigenfrequency $\left[(\Delta+\alpha V_{\text{pump}}^2/2)^2-(\alpha V_0 V_{\text{pump}})^2\right]^{-1/2}$ when $\alpha V_0 V_{\text{pump}} < |\Delta+\alpha V_{\text{pump}}^2/2|$. This leads to the rapid change in the measured peak frequency below the oscillation threshold (Fig. 3f).

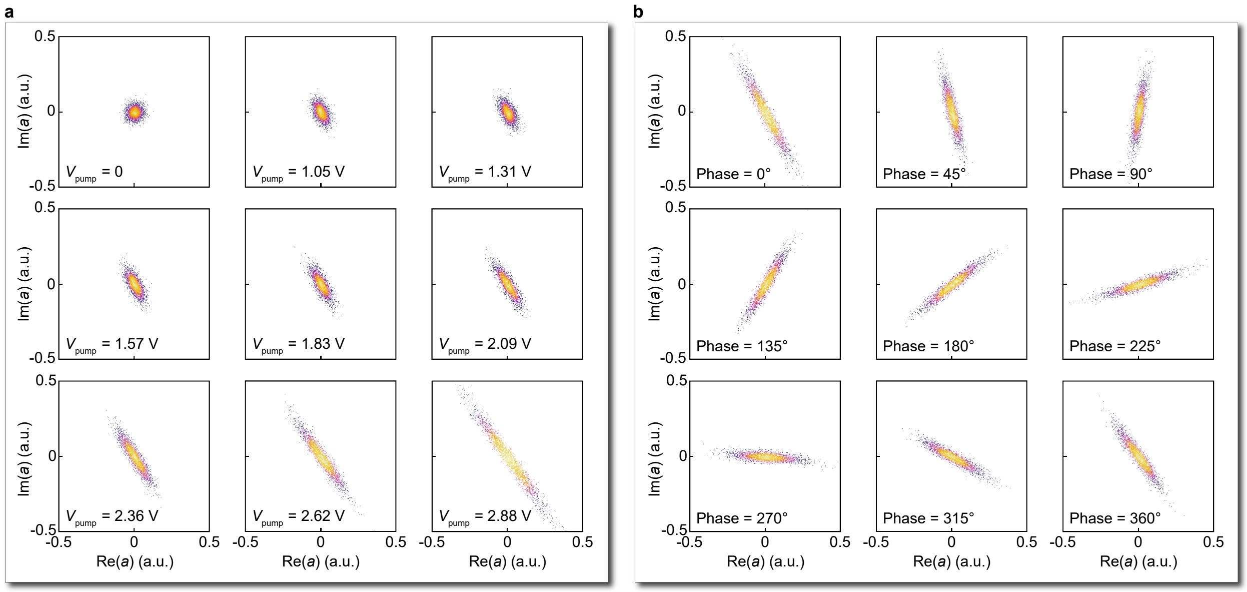

Figure S4. Squeezed quadrature of the nanoelectromechanical Dirac-vortex states. **a**, Measured vibration quadrature of the Dirac-vortex state under different pump voltages. **b**, Measured vibration quadrature of the Dirac-vortex state under different pump phases with a fixed pump voltage V_{pump} = 2.62 V. All the experimental results were obtained from a device with R_0/l_0 = 0.5 under a pump frequency $2\Omega = 2\omega_0$. A weak white-noise voltage with center frequency ω_0 and frequency bandwidth 50 kHz was used to directly actuate the device.

4. Parametric oscillation behavior of the Dirac-vortex states

The influence of external electrical drive has been discussed in the previous section. Actually, the nanomechanical Dirac-vortex state also exhibits strong third-order nonlinearity, where a nonzero phonon number

$\hat{a}^{\dagger}\hat{a}$ leads to a frequency shift of the Dirac-vortex mode itself. The classical dynamics of the Dirac-vortex state is governed by an autonomous differential equation

$$\frac{\partial a(t)}{\partial t} = -j\left[\Delta + \alpha V_{\text{pump}}^2/2 - \beta|a(t)|^2\right]a(t) - j\alpha V_0 V_{\text{pump}} a^*(t) - \gamma a(t), \tag{S24}$$

where β is the third-order nonlinear coefficient, and γ is the damping rate of the Dirac-vortex state. We assume $a(t) = A(t)e^{-j\Phi(t)}$ so that Eq. (S24) can be rewritten as

$$\begin{cases} \dfrac{\partial A}{\partial t} = \left(-\gamma + \alpha V_0 V_{\text{pump}} \sin(2\Phi)\right)\cdot A, \\ \dfrac{\partial \Phi}{\partial t}\cdot A = \left(\Delta + \alpha V_{\text{pump}}^2/2 - \beta A^2 + \alpha V_0 V_{\text{pump}} \cos(2\Phi)\right)\cdot A. \end{cases} \tag{S25}$$

In the steady state, we have $\frac{\partial A}{\partial t} = 0$ and $\frac{\partial \Phi}{\partial t} = 0$, so we obtain the nonzero steady-state solution of Eq. (S25)

$$\begin{cases} A = \left(\dfrac{\Delta + \alpha V_{\text{pump}}^2/2 + \alpha V_0\sqrt{V_{\text{pump}}^2 - V_{\text{th}}^2}}{\beta}\right)^{1/2}, \\ \Phi = \dfrac{\arcsin\left(V_{\text{th}}/V_{\text{pump}}\right)}{2}, \end{cases} \tag{S26}$$

where $V_{\text{th}} = \gamma/\alpha V_0$ is the minimal threshold pump voltage of the parametric phonon oscillator. Note that Eq. (S24) always has a zero steady-state solution $a(t) = 0$. The phonon oscillation requires this zero solution to be unstable such that a slight quantum fluctuation near this point can always lead the system to the nonzero steady-state solution in Eq. (S26). To analyze the stability of the zero solution, we rewrite Eq. (S24) as a set of linear autonomous equations

$$\begin{pmatrix} d\,\text{Re}(a)/dt \\ d\,\text{Im}(a)/dt \end{pmatrix} = \begin{bmatrix} -\alpha V_0 V_{\text{th}} & \Delta + \alpha V_{\text{pump}}^2/2 - \alpha V_0 V_{\text{pump}} \\ -\Delta - \alpha V_{\text{pump}}^2/2 - \alpha V_0 V_{\text{pump}} & -\alpha V_0 V_{\text{th}} \end{bmatrix} \begin{pmatrix} \text{Re}(a) \\ \text{Im}(a) \end{pmatrix}.$$

Here, we have ignored the third-order nonlinearity term, because we consider the dynamics near $a = 0$ only. To ensure that the zero solution is unstable, at least one of the eigenvalues of the 2×2 matrix in the above equation must be positive, so the following condition must be satisfied:

$$V_{\text{pump}}^2 > \left(\Delta/\alpha V_0 + V_{\text{pump}}^2/2V_0\right)^2 + V_{\text{th}}^2. \tag{S27}$$

In conclusion, as long as Eq. (S27) is satisfied, one can experimentally observe parametric phonon oscillation with amplitude and phase governed by Eq. (S26). With Eq. (S27), one can also obtain the lowest threshold pump voltage $V_{\mathrm{pump}} = V_{\mathrm{th}}$ when the frequency detuning is $\Delta = -\alpha V_{\mathrm{th}}^2/2$.

Figure S5 presents the theoretically calculated phonon oscillation intensity under a coherent pump. To fit the experimental results in Fig. 2b, we used the following parameters: the minimal threshold pump voltage V_{th} = 2.09 V, the Kerr nonlinear coefficient β = 0.392 kHz nm^{-2}, and the electromechanical tuning coefficient α = 0.40 kHz V^{-2}. Note that we adopted the resonant frequency $\omega_0/2\pi$ = 45.217 MHz during the theoretical fitting, which is slightly different from the measured resonant frequency $\omega_0/2\pi$ = 45.238 MHz in Fig. 1e. With these parameters, we could estimate the damping rate of the Dirac-vortex state as $\gamma/2\pi$ = 8.4 kHz, which agrees well with the fitted value of $\gamma/2\pi$ = 7.9 kHz for the measured results in Fig. 1e.

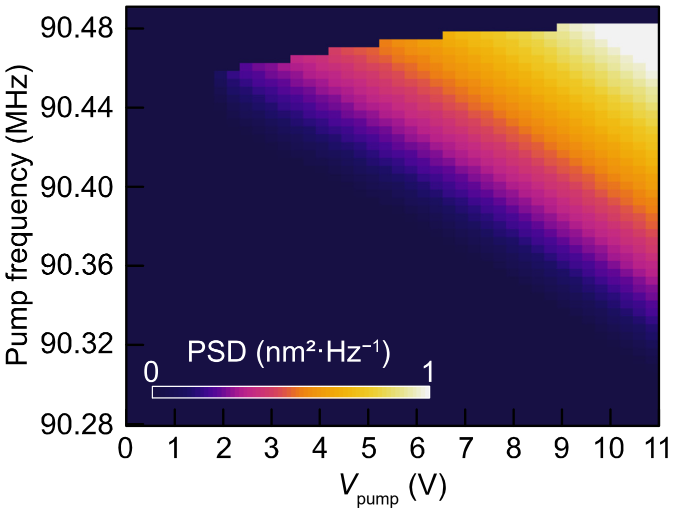

Figure S5. Theoretically calculated parametric phonon oscillation intensity under a coherent pump.

5. Topological parametric amplification of mechanical signals

In this section, we analyze the topological parametric amplification of nanomechanical Dirac-vortex states under an external drive. In our experiment, the applied voltage is $V = V_0 + V_{\mathrm{signal}}\sin(\Omega t + \phi) + V_{\mathrm{pump}}\sin(2\Omega t)$, which consists of a d.c. voltage V_0, an a.c. signal voltage with amplitude V_{signal} and frequency Ω for direct actuation of mechanical motion, and an a.c. pump voltage with amplitude V_{pump} and frequency 2Ω. Here Ω is set near the resonant frequency ω_0 of the Dirac-vortex state. Considering that $W(\mathbf{r}, t)$ is far less than the distance d between the electrode and the ground, Eq. (S1) can be rewritten as

$$\rho h\frac{\partial^2 W(\mathbf{r},t)}{\partial t^2}+D\nabla^4 W(\mathbf{r},t)\cdot\zeta(\mathbf{r})=\frac{\varepsilon\left(V_0+V_{\text{signal}}\sin(\Omega t+\phi)+V_{\text{pump}}\sin(2\Omega t)\right)^2}{2d^2}$$
$$+\frac{\varepsilon\left(V_0+V_{\text{signal}}\sin(\Omega t+\phi)+V_{\text{pump}}\sin(2\Omega t)\right)^2}{d^3}W(\mathbf{r},t). \tag{S28}$$

Assuming $W(\mathbf{r}, t) = a(t)f_0(\mathbf{r})e^{j\Omega t}$ + h.c. and retaining the terms containing $e^{j\Omega t}$, we obtain

$$\rho h\left[2j\Omega\frac{\partial a(t)}{\partial t}-\left(\Delta^2+2\omega_0\Delta+\frac{\varepsilon}{\rho h d^3}V_0^2+\frac{\varepsilon}{2\rho h d^3}V_{\text{pump}}^2\right)a(t)+j\frac{\varepsilon}{\rho h d^3}V_0V_{\text{pump}}a^*(t)\right]f_0(\mathbf{r})$$
$$=\frac{-j\varepsilon V_0V_{\text{signal}}e^{j\phi}}{2d^2}.$$

Next, we define a parameter $\chi=\varepsilon\int f_0(\mathbf{r})d\mathbf{r}\big/(4\omega_0 m_{\text{eff}}d^2)$ with $m_{\text{eff}}=\rho h\int\left(f_0(\mathbf{r})\right)^2 d\mathbf{r}$ being the effective mass of the nanomechanical Dirac-vortex state. Taking into consideration the damping rate γ of the Dirac-vortex state, the equation can be rewritten as

$$\frac{\partial a(t)}{\partial t}=-j\bar{\Delta}a(t)-\alpha V_0V_{\text{pump}}a^*(t)-\gamma a(t)-\chi V_0V_{\text{signal}}e^{j\phi}, \tag{S29}$$

with the effective detuning $\bar{\Delta}=\Delta+\alpha V_{\text{pump}}^2\big/2$. In the steady state $\partial a(t)/\partial t=0$, Eq. (S29) has a solution

$$a=\frac{-\chi V_0V_{\text{signal}}\left[\alpha V_0V_{\text{pump}}e^{-j\phi}+(j\bar{\Delta}-\gamma)e^{j\phi}\right]}{(j\bar{\Delta}+\gamma)(j\bar{\Delta}-\gamma)+(\alpha V_0V_{\text{pump}})^2}. \tag{S30}$$

Therefore, the gain of the topological amplifier is

$$G(\phi)=\frac{|a|_{\text{pump on}}}{|a|_{\text{pump off}}}=\left|\frac{\left[\alpha V_0V_{\text{pump}}e^{-2j\phi}+(j\bar{\Delta}-\gamma)\right](j\bar{\Delta}+\gamma)}{(j\bar{\Delta}+\gamma)(j\bar{\Delta}-\gamma)+(\alpha V_0V_{\text{pump}})^2}\right|. \tag{S31}$$

In the special case of a zero detuning $\bar{\Delta}=0$, the parametric gain is

$$G(\phi)=\left[\frac{\cos^2\phi}{(1+V_{\text{pump}}/V_{\text{th}})^2}+\frac{\sin^2\phi}{(1-V_{\text{pump}}/V_{\text{th}})^2}\right]^{1/2} \tag{S32}$$

where $V_{\text{th}}=\gamma/\alpha V_0$ is the threshold pump voltage of the parametric phonon oscillator.

Figure S6 presents the measured and theoretically predicted parametric amplification of the nanoelectromechanical Dirac-vortex states. The experimental results were obtained from a device with R_0/l_0 = 0.5 under a pump frequency $2\Omega/2\pi$ = 90.472 MHz. Note that we adopted the resonant frequency $\omega_0/2\pi$ = 45.222

MHz during the theoretical fitting, which is slightly different from the measured resonant frequency $\omega_0/2\pi$ = 45.238 MHz in Fig. 1e.

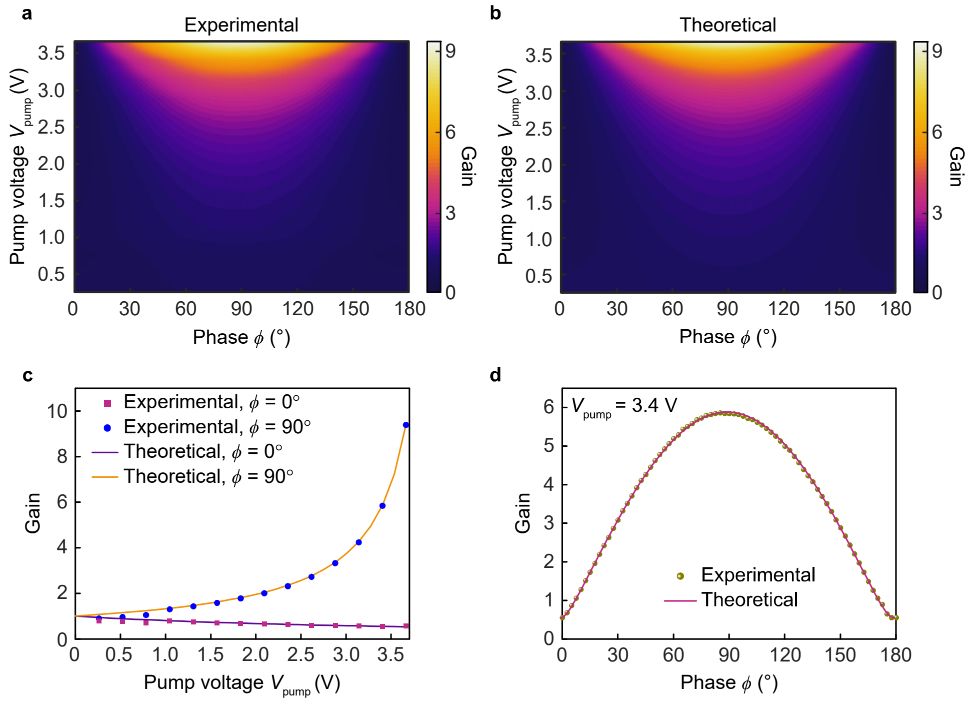

Figure S6. Parametric amplification of the nanoelectromechanical Dirac-vortex states. **a**, **b**, Experimentally measured (**a**) and theoretically calculated (**b**) parametric gain of the mechanical signal $a(t)$ as a function of the phase and voltage of a coherent pump. The parametric gain is defined as $|a|_{\text{pump on}}/|a|_{\text{pump off}}$. **c**, Parametric gain of the mechanical signal as a function of the pump voltage V_{pump} with the pump phase ϕ fixed at 0° or 90°. **d**, Parametric gain of the mechanical signal as a function of the pump phase with the pump voltage V_{pump} fixed at 3.4 V.

6. Additional data

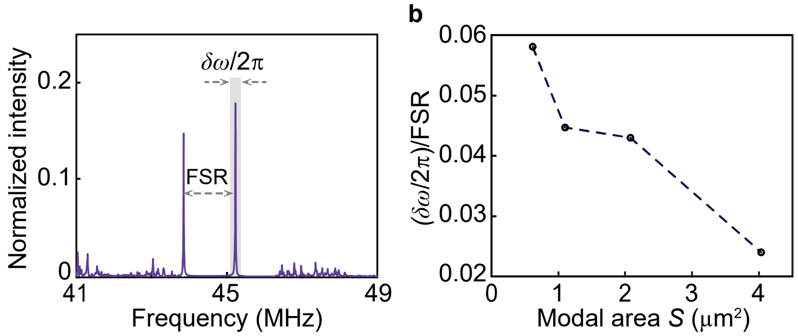

Figure S7. **a**, Measured mechanical intensity spectrum of a device. FSR denotes the free spectral range, and $\delta\omega/2\pi$ denotes the standard deviation of resonant frequency for different devices with the same designed structural parameters. **b**, Experimentally measured relationship between $(\delta\omega/2\pi)$/FSR and modal area S.

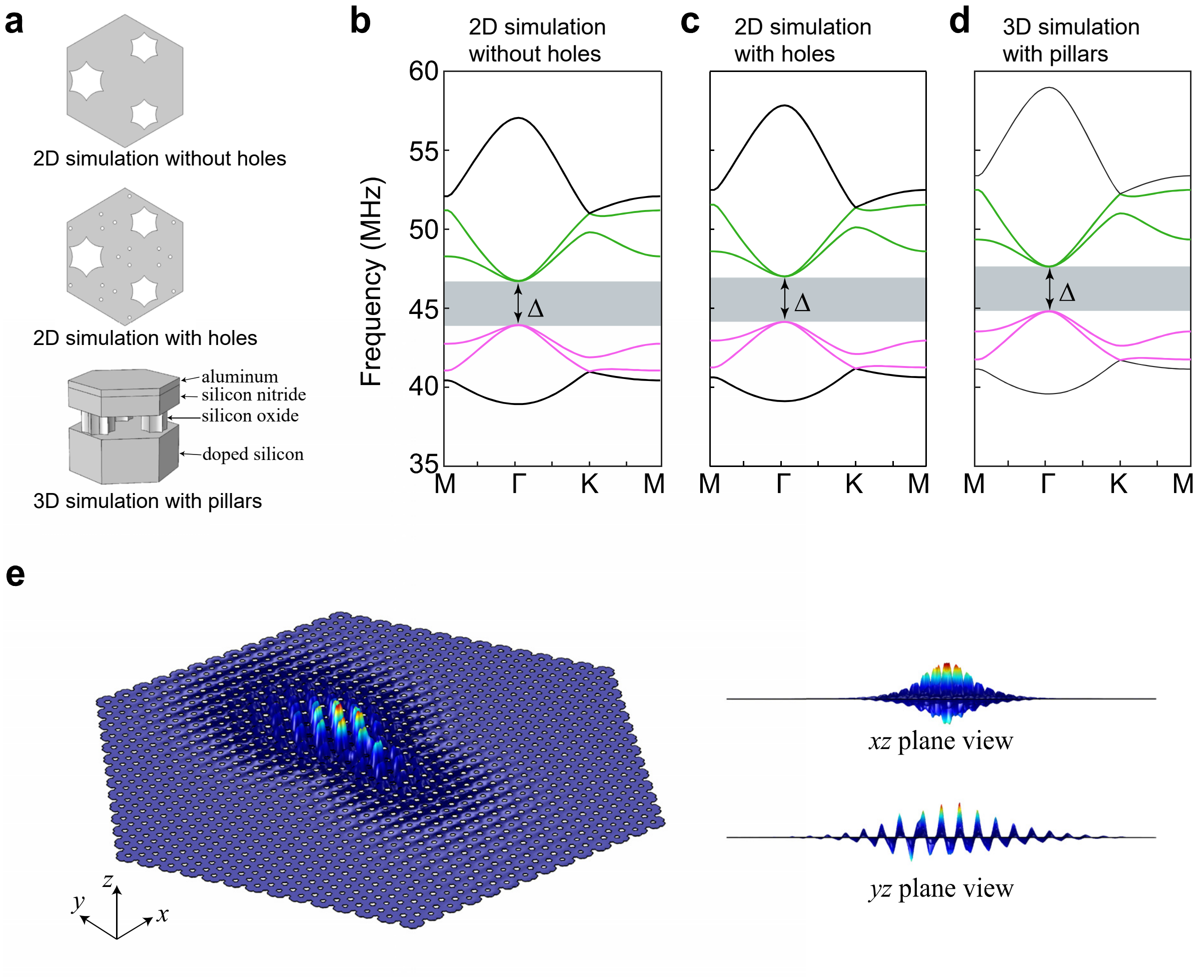

Figure S8. Effect of holes and pillars on bulk energy band diagram and simulated modal profile of the Dirac-vortex state. **a**, Geometries of the simulated crystal unit cell in 2D simulation without the holes, in 2D simulation with the holes, and in 3D simulation with the supporting oxide pillars (the thickness of each layer is enlarged 25 times for clarity). For convenient setting of boundary conditions in the simulation, the unit cell is the same as that in Fig. 1b but shifted along the horizontal direction by $l_0/3$. The geometric parameters of the crystal unit cell are $\theta = \pi/2$ and δ_0 = 300 nm. **b–d**, Simulated bulk energy band diagrams for the nanomechanical crystal structure under the three simulation settings in **a**. A comparison between the simulated results in **b** and **c** concludes that the holes in the membranes only slightly shift the frequency of the bulk bands. A comparison between the simulated results in **b** and **d** concludes that the pillars in the membranes also only slightly shift the frequency of the bulk bands without affecting the topological bulk band structure. It is also found that the pillars introduce some loss to the bulk states because they serve as energy leakage channels to the silicon substrate. **e**, Modal profile of the Dirac-vortex state in 3D, *xz*-plane, and *yz*-plane views.
